# Contemporary employment arrangements and mental well-being in men and women across Europe: a cross-sectional study

**DOI:** 10.1186/s12939-014-0090-6

**Published:** 2014-10-28

**Authors:** Deborah De Moortel, Hadewijch Vandenheede, Christophe Vanroelen

**Affiliations:** Department of Sociology, Vrije Universiteit Brussel, Pleinlaan 2, 1050 Brussel, Belgium; Health Inequalities Research Group. Employment Conditions Knowledge Network (GREDS\Emconet), Universitat Pompeu Fabra, Plaça de la Mercè 10-12, 08002 Barcelona, Spain

**Keywords:** Employee well-being, Europe, Gender inequalities, Employment quality, Welfare regimes

## Abstract

**Introduction:**

There is the tendency in occupational health research of approximating the ‘changed world of work’ with a sole focus on the intrinsic characteristics of the work task, encompassing the job content and working conditions. This is insufficient to explain the mental health risks associated with contemporary paid work as not only the nature of work tasks have changed but also the terms and conditions of employment. The main aim of the present study is to investigate whether a set of indicators referring to quality of the employment arrangement is associated with the well-being of people in salaried employment. Associations between the quality of contemporary employment arrangements and mental well-being in salaried workers are investigated through a multidimensional set of indicators for employment quality (contract type; income; irregular and/or unsocial working hours; employment status; training; participation; and representation). The second and third aim are to investigate whether the relation between employment quality and mental well-being is different for employed men and women and across different welfare regimes.

**Methods:**

Cross-sectional data of salaried workers aged 15–65 from 21 EU-member states (n =11,940) were obtained from the 2010 European Social Survey. Linear regression analyses were performed.

**Results:**

For both men and women, and irrespective of welfare regime, several sub-dimensions of low employment quality are significantly related with poor mental well-being. Most of the significant relations persist after controlling for intrinsic job characteristics. An insufficient household income and irregular and/or unsocial working hours are the strongest predictors of poor mental well-being. A differential vulnerability of employed men and women to the sub-dimensions of employment quality is found in Traditional family and Southern European welfare regimes.

**Conclusions:**

There are significant relations between indicators of low employment quality and poor mental well-being, also when intrinsic characteristics of the work task are controlled. Gender differences are least pronounced in Earner-carer countries.

## Introduction

At the end of the 20th century, the nature of work-related health risks shifted in Europe. In general, physical exposures, dangerous and physically hard labour, which characterised industrial production, became relatively less important, while the psychosocial work environment became a primary source of work-related health risks. In occupational health research, studies using conceptual frameworks such as the Job Demand-Control-Support (DCS) model [1,2], the Effort-Reward Imbalance model [3] or the Job Demand-Resources model [4] emerged, putting the relationship between psychosocial job characteristics and employee well-being high on the research agenda [5]. A positive and sizeable association between adverse psychosocial working conditions and both mental ill health and mental distress has been shown [6,7]. For instance, studies using the DCS model have revealed that high psychological demands have a detrimental effect on mental health [8], while a high degree of job control is related to better mental health [9]. However, by applying standard psychosocial risk models, there is a tendency in research of approximating the ‘changed world of work’ with a sole focus on the intrinsic characteristics of the work task (i.e. the job content and working conditions) [10]. This is arguably insufficient for explaining the entirety of mental health risk associated with contemporary paid work as not only the nature of work tasks has changed but also the terms and conditions of employment [11]. In contemporary labour markets, market risks are increasingly transferred to the workforce in the form of flexible employment arrangements (e.g. atypical contracts, working hours flexibility, ‘small jobs’, etc.). These new types of employment cannot be considered ‘neutral’ in terms of their impact on workers’ mental well-being [12-14].

In epidemiological research, concepts as ‘precarious’, ‘non-standard’ or ‘contingent’ employment are used in order to understand the potential health consequences of ‘*the quality of employment’* [13,15]. These contemporary employment arrangements are then defined by the absence of features characterising the ‘old’ Fordist Standard Employment Relationship (SER): i.e. a long-term commitment between the employee and the employer, in which full-time, permanent employment is combined with regular work schedules and a range of benefits and entitlements [16].

Studies furthermore show that women are overrepresented in these new types of employment, highlighting the need for further research into this potential source of excess vulnerability to poor mental well-being in employed women [17,18].

In summary, complementing indicators of the intrinsic quality of the work task with indicators of the quality of the prevailing employment arrangements allows for capturing the contemporary work situation in a more complete manner. In this article, a set of indicators representing ‘employment quality’, are related to mental well-being. The main aim of the present study is to investigate whether indicators for the quality of the employment arrangement are associated with employee well-being – and whether this association holds after controlling for the intrinsic characteristics of the work task. The second and third aim are to investigate whether the relation between employment quality and mental well-being is different for employed men and women and across European welfare regimes.

### Contemporary employment arrangements and mental well-being

Unravelling the association between employment quality and mental well-being is a challenging task, as consensus on how to measure employment quality has not yet been reached*.* Conceptually, employment quality has previously been addressed in different ways. In most cases, single measurement items (such as job insecurity or non-permanent employment) were used [19,20]. More recently, a multidimensional approach is becoming more common as contemporary employment arrangements tend to deviate in different respects from the ‘gold standard’ of life-long full-time SER-employment [21].

The indicators of employment quality applied in this study are selected on the basis of a multidimensional concept proposed by Benach and colleagues [22]. This multidimensional concept was developed in order to explore the potential relations between employment quality and outcomes of worker health and well-being through proxy-indicators of the employment quality dimensions available in large-scale quality of work surveys [22]. Benach and colleagues propose a model for employment quality encompassing two conceptual dimensions: (1) employment conditions and (2) employment relations. For each conceptual dimension, several sub-dimensions are defined: four for employment conditions (contract security, working times, income and rights, and employability) and two for employment relations (empowerment and representation). If an employee reports the most favourable situation for each sub-dimension, the employment arrangement is similar to the gold standard of SER-employment. However, the flexible labour market generates a series of different non-standard employment arrangements. Hence, most contemporary employment arrangements are characterised by a combination of standard and non-standard or flexible arrangements [21]. Previous research has already probed into the relation of many of the above-described sub-dimensions of employment quality with mental health, but has never examined all sub-dimensions simultaneously in a large representative cross-national sample of the general working population.

### Gender distribution of low employment quality

In general, the quality of employment tends to be lower among employed women [14,15]. A study based upon the data of the Fourth European Working Conditions Survey (2005) investigated the gender distribution of low employment quality in the EU-27 and revealed that women are less likely than men to have permanent contracts, are more often holding low paid jobs, have fewer non-wage benefits, are less likely to be involved in decision-making and are overrepresented in voluntary and involuntary part-time employment [17]. On the other hand, a low degree of control over working hours, uncompensated flexible working hours and long working hours are more common for men than for women [17]. Even when men and women have the same job titles, they tend to face different quality of employment [23]. For instance, female managers are often paid less, receive fewer non-wage benefits, have less freedom to determine their work schedule and have fewer training opportunities, compared to male managers [17]. Explanations for these gender inequalities are, amongst other, to be found in theories of roles and role sets. Social structural forces sort people into different roles based upon their sex [23]. Even in contemporary society the ‘unwritten gender contract’, assigning men a primary role in breadwinning and women central responsibility for care-giving, remains strong [15]. Although both men and women place high value on both employee and homemaker roles [24], women are more likely to experience agency poverty. That is: a lower degree of freedom to choose between alternative sets of accomplishments [25]. The gender division of labour dictates that even when women enter employment, they typically maintain the main responsibility in the family for childcare and housework [26].

Agency inequality is, however, affected by welfare state policies and institutional models [25]. Korpi et al. [27] analysed the consequences of family policies on gender inequality outcomes. In Europe, three types of policy models can be distinguished based on the extent to which legislations and policies are supportive for mothers’ role in paid work or in traditional homemaking: (1) the Earner-carer model (Nordic countries), which facilitates women’s full-time employment and continuous paid work; (2) the Traditional family model (Continental Europe), which supports families by facilitating part-time work for women; and (3) the Market-oriented policy model (Anglo-Saxon countries), largely leaving it to parents to solve problems of social care by relying on market services [27,28]. Policies promoting women’s employment, such as Earner-carer family policies, have a positive effect on women’s economic opportunities and mitigate the adverse effects on well-being attributed to stressful working conditions [27]. In this study the relation between employment quality and mental well-being will be studied across gender and welfare regime using the typology of Korpi et al. [27]. This welfare regime typology is relevant for gender inequality research as it considers gender inequality both in terms of material inequality and in terms of inequalities with respect to capabilities to choose over a range of alternatives [27]. The Southern European and Eastern European (or Contradictory) welfare regimes are also included as they are increasingly analysed as separate welfare regimes [29]. A brief definition of the policy models is available in Table 1.

**Table 1:** **European welfare regimes, categorised by family policies [** 27 **]**

**Welfare regime**	**Countries**	**N (Non-weighted)**		**Characteristics**
		**Men**	**Women**	
**Earner-carer**				
	Denmark	338	320	Policies facilitate women’s full-time employment and continuous engagement in paid work. Female labour force participation, particularly that of mothers, is encouraged by transferring major parts of care from the home to the public sector.
	Finland	339	355	
	Sweden	312	343	
	*Subtotal*	*989*	*1,018*	
**Traditional family**				
	Belgium	317	335	Policies support families by supporting women’s unpaid work within the home. It is presumed that women have the main responsibility for care at home and enter paid work primarily as secondary earners.
	Germany	659	527	
	France	331	372	
	The Netherlands	341	368	
	*Subtotal*	*1,648*	*1,602*	
**Southern European**				
	Cyprus	72	66	This policy model shows similarities with the Traditional family model, however the average social expenditure on family and children is very low [30].
	Spain	345	296	
	Greece	199	235	
	Portugal	185	222	
	*Subtotal*	*801*	*819*	
**Market-oriented**				
	United Kingdom	357	441	Policies are characterized by the absence of strong state intervention to support earner-carer or traditional households. Parents have to solve problems of social care by relying on market services.
	Ireland	213	296	
	*Subtotal*	*570*	*737*	
**Contradictory**				
	Czech Republic	338	301	This policy model is characterized by emphasize on both traditional family values (a traditional division of housework) and high female participation in paid work [31].
	Estonia	278	381	
	Hungary	242	245	
	Lithuania	102	190	
	Poland	270	234	
	Bulgaria	260	338	
	Slovenia	208	229	
	Slovakia	203	268	
	*Subtotal*	1,901	2,186	
***Total***		***5,909***	***6,362***	

### Aims and hypotheses

The central focus of this study is to investigate whether low employment quality is negatively associated with employee mental well-being. It is also tested whether this association is not confounded by low quality of intrinsic characteristics of the work task given that employees in a job with a higher overall intrinsic work quality also tend to experience higher employment quality [22]. Nevertheless, we hypothesize that the association between employment quality and well-being remains after statistical control for the intrinsic characteristics of the work task. To our knowledge, no study thus far has documented whether low employment quality, measured as a multidimensional set of indicators, is negatively associated with employee well-being (independent from intrinsic work characteristics). Moreover, as women more often need to balance the demands of paid and unpaid labour, a higher vulnerability to the sub-dimensions of employment quality can be expected among women. Furthermore, based upon the research of Korpi et al. [27] we expect that family policy models are (partially) able to mediate this differential vulnerability through policies facilitating work-family reconciliation. Therefore, we expect a differential vulnerability of men and women in all welfare regimes, except for the Earner-carer model.

## Methods

### Data

Data from the European Social Survey (ESS) 2010 was used. The ESS is a European cross-national survey that is conducted bi-annually since 2001. The ESS 2010 includes representative samples of persons aged 15 and over, who are resident in one of 27 European countries. Data was collected through face-to-face interviews including questions reoccurring in every round of ESS and questions from an ESS-2010-specific module on Work, Family and Well-being. Due to the exclusive use of secondary data (ESS data), which is available to the public, no ethical approval is required for this study. This study focuses on wage earners in the 21 European Union member states included in the ESS 2010 (Belgium, Bulgaria, Cyprus, Czech Republic, Denmark, Estonia, Finland, France, Germany, Greece, Hungary, Ireland, Lithuania, Netherlands, Poland, Portugal, Slovakia, Slovenia, Spain, Sweden and United Kingdom). All respondents from non-EU countries, not in waged employment or older than 65, were excluded from the analyses. The total sample consisted of 7,119 male and 6,988 female employees. Most countries had a sufficient response rate (range between 39.4% and 76.1%), with the exception of Germany having a response rate of 29.7%. The ESS data is widely used and shows sample distributions that are comparable with other databases [32,33].

### Variables

#### Dependent variable

Poor mental well-being was measured by three items from the WHO-5 Well-being Index [34]. The WHO-5 Well-being Index is a measure of positive affect [35]. The ESS 2010 only contained three of the original five items of the WHO-5 Well-being Index [35]. However, the internal consistency of the Well-being index has proven to be excellent. The three items have a Cronbach’s alpha of 0.81 across the whole ESS 2010 sample and a Cronbach’s alpha of 0.79 across the study sample, which is only marginally lower than the Cronbach’s alpha of 0.82 found across the whole ESS 2004 sample, which contained all five items from the WHO-5 Well-being index [34]. Consequently, we can be confident that the use of the three-item scale does not lead to different results. The questions included were: (1) ‘Over the last two weeks I have felt cheerful and in good spirits’, (2) ‘Over the last two weeks I have felt calm and relaxed’, (3) ‘Over the last two weeks I have felt active and vigorous’. Answers are coded from 1 to 6 ranging from ‘All of the time’ to ‘At no time’ [35]. The item scores were summed up and then normalised to a 0 to 10 range. Whenever an item on the dependent variable was missing, this item was attributed a value using expectation-maximisation as imputation method [36].

#### Independent variables

Seven proxy indicators were constructed for measuring low employment quality. A brief definition of the dimensions and a description of the construction method applied for creating the proxy indicators of employment quality are available in Table 2.

**Table 2:** **Construction of proxy indicators for employment quality in ESS 2010**

**Dimension**	**Proxy indicator**	**Source indicator(s)**	**Scoring**
**Employment conditions**			
**Contract security**	Type of employment contract	Type of employment contract	1) Permanent
Reflects the degree of certainty of continuing work.			2) Non-permanent
**Income and rights**	Income	Combination of:	1) Sufficient household income
Amount of pay and social rights (e.g. sickness insurance) or fringe benefits derived from employment.	*(no proxy for social rights and benefits available)*	a- Living comfortably on present household income?	2) Contributory earner with insufficient household income
		b - Around how large a proportion of the household income do you provide yourself?	3) Main earner with insufficient household income
**Working hours**	Employment status	Combination of:	1) Full-time (>35 hours)
Features of the working times are working long hours, working non-fixed day shifts, weekend work, having variable daily working hours, working evenings and nights.		a- Total hours normally worked per week in main job overtime included?	2) Part-time
		b- How many hours would choose to work weekly?	3) Involuntary part-time
	Irregular and/or unsocial working hours	Combination of:	An indicator for unsocial working hours was created, combining ‘working weekends’ with ‘working evenings/nights’. The indicator for unsocial working hours was added to indicators for ‘working overtime at short notice’ and ‘intensive working hours’, resulting in an overall indicator for irregular and/or unsocial working hours. The variables were normalised to range from 0 to 10, with 10 being the least-favourable situation (Cronbach’s alpha = 0.60).
		a- Work involves working weekends	
		b- Work involves working nights/evenings	
		c- Have to work overtime at short notice	
		d- Intensive working hours	
**Employability**	Training	Having been on a course for work during the last 12 months?	1) No
Reflects the capability of maintaining employment in the future.			2) Yes
**Employment relations**			
**Representation**	Representation	Regular meetings between representatives of the employees and employers, in which working conditions and practices can be discussed?	1) No
Having a collective voice (e.g. the presence of a trade union).			2) Yes
**Empowerment**	Participation	Possibility for employees to influence policy decisions?	The variable ranges from 0 to 10, with 10 being the least-favourable situation.
Practices regarding employee participation in problem solving and decision making.			

Countries were grouped according to an adaptation of the family policy typology of Korpi et al. [27]. This typology is used as it assesses the impact of different (institutionalised) policies which could be hypothesised to affect agency inequality between men and women at the individual level. We expanded the original typology to include two more country types: the Southern European and Eastern European (or Contradictory) countries [31]. The country classifications are available in Table 1.

#### Control variables

Low quality of intrinsic characteristics of the work task was measured by the DCS model [1], as well as an indicator representing the lack of career opportunities. The latter indicator is included because of its inherent relation with the content of the work task. Sum scales for low skill discretion and low autonomy were created. *Low skill discretion* (Cronbach’s alpha=0.67) is measured by three items: (1) ‘variety in work’; (2) ‘job requires learning new things’, and (3) ‘how long for somebody with the right qualifications to learn to do your job well’. The *low autonomy* scale (Cronbach’s alpha=0.70) also consists of three items: (1) ‘allowed to decide how daily work is organised’; (2) ‘can decide time start/finish work’; and (3) ‘allowed to choose/change pace of work’. *High psychological demands* are based on one five-point Likert scale: ‘I have never enough time to get everything done in my job’. *Lack of co-worker support* was assessed using the following question: ‘In the current job: I can get support/help from my co-workers when needed’ and response categories were ‘not at all true’, ‘a little true’, ‘quite true’ and ‘very true’. This indicator was dichotomised, with the category ‘not at all true’ being considered as the low support group. Finally, *lack of career opportunities* is measured by the degree of ‘opportunities for advancement’ in the current job, assessed by one five-point Likert scale. Whenever an item was missing on the low skill discretion and the low autonomy scale, this item was attributed a value using expectation-maximisation as imputation method [36]. The scales for low skill discretion, low autonomy, high psychological demands and lack of career opportunities were normalised to a range from 0 to 10, with 10 being the least-favourable situation [37].

Other control variables, included in the models, are: education (low, medium or high), age (15–29, 30–49 or 50–65), children living at home (0, 1 or ≥2) and single parent (yes/no). The employees were grouped into three educational categories according to the International Standard Classification of Education (ISCED): ‘low’ (up to lower secondary); ‘medium’ (up to post-secondary non-tertiary); and ‘high’ (completed tertiary education). Age was recoded into three age groups corresponding with three main periods in a working career: lift-off (15–29 years), a mid-career period (30–49 years), and the end-of-career period (50–65 years) [38].

#### Statistical analyses

To describe the sample, percentages, means, and standard deviations were used. To examine the association between the indicators of employment quality and mental well-being, linear regression models were estimated for men and women separately. Firstly, two models were estimated with the individual background variables and the indicators for employment quality (Model 1) and quality of intrinsic characteristics of the work task (Model 2) added as blocks. Secondly, a full model was estimated (Model 3), including all covariates. To probe into differences in the vulnerability to low employment quality for employed men and women, an additional model was estimated for men and women jointly, taking the interactions between gender and the indicators for employment quality into account (Model 4). To examine cross-country differences in the association between employment quality and mental well-being, Model 3 and 4 were also estimated as stratified by welfare regime. At all steps, parameter effects of the covariates in relation with poor mental well-being are presented as beta estimates, with their related standard errors. For all models, R-squared estimates of model strength are shown. Throughout the analyses, data have been weighted by population weights that correct for population size and by design weights that correct for chances of unequal selection probability. All analyses were performed using SPSS version 20. The final sample consisted of 6,071 male and 5,869 female employees (with list-wise deletion of non-imputed missing data).

## Results

### Descriptive results

Means and standard deviations for the continuous variables and percentages for the categorical variables are presented in Table 3. Across all welfare regimes, men report a better mental well-being than women. Women report higher levels of education and are more often single parents compared to men. As for employment quality, women less often state to have a sufficient income and have higher rates of involuntary part-time employment compared to men. In all welfare regimes, there are generally high levels of sufficient income, except in Southern European and Contradictory welfare regimes.

**Table 3:** **Sample characteristics and descriptive statistics (percentages) stratified by welfare regime and gender**

	**Traditional family**		**Southern European**		**Contradictory**		**Earner-carer**		**Market-oriented**	
	**Men**	**Women**	**Men**	**Women**	**Men**	**Women**	**Men**	**Women**	**Men**	**Women**
	**n = 3,044**	**n = 2,785**	**n = 870**	**n = 811**	**n = 965**	**n = 938**	**n = 340**	**n = 355**	**n = 852**	**n = 980**
**DEMOGRAPHIC AND HEALTH CHARACTERISTICS**										
**Poor mental well-being** ^**1**^	3.3 (1.9)	3.6 (2.1)	2.8 (1.7)	3.4 (1.8)	3.4 (2.1)	3.6 (2.1)	3.1 (1.7)	3.4 (1.7)	3.2 (2.0)	3.8 (2.1)
**Age**										
15-29 years old	15.2	16.5	14.2	19.2	25.2	17.7	14.3	13.1	22.4	16.5
30-49 years old	53.8	53.4	62.6	58.2	48.6	54.3	52.9	52.6	50.2	54.9
50-65 years old	31.0	30.1	23.2	22.6	26.2	27.9	32.8	34.3	27.4	28.6
**Education**										
Low	11.2	12.1	42.0	36.5	6.8	4.8	11.3	7.5	22.8	21.8
Medium	55.1	51.9	26.6	22.9	69.6	57.2	47.6	40.9	36.5	31.6
High	33.7	36.1	31.4	40.6	23.5	38.0	41.1	51.6	40.8	46.6
**Children living at home**										
0	50.9	47.7	47.0	46.8	44.1	37.5	51.9	49.7	51.4	41.8
1	19.2	24.2	20.7	26.1	28.2	29.0	18.8	18.4	18.6	26.2
≥ 2	29.9	28.1	32.3	27.1	27.6	33.5	29.3	31.9	30.1	32.0
**Single parent**										
No	97.5	89.3	99.2	91.4	97.9	86.9	97.4	91.8	96.0	86.5
Yes	2.5	10.7	0.8	8.6	2.1	13.1	2.6	8.2	4.0	13.5
**EMPLOYMENT QUALITY**										
**Type of contract**										
Permanent	88.0	88.1	80.8	78.1	79.2	79.1	92.0	88.8	92.4	88.6
Non-permanent	12.0	11.9	19.2	21.9	20.8	20.9	8.0	11.2	7.6	11.4
**Income**										
Sufficient income	87.9	88.1	79.0	79.6	72.4	67.8	95.4	94.9	91.3	82.0
Contr. earner, insuf. inc.	2.9	5.5	4.3	12.5	10.4	19.4	1.1	1.8	2.4	9.8
Main earner, insuf. inc.	9.2	6.4	16.7	7.8	17.2	12.8	3.5	3.3	6.3	8.2
**Employment status**										
Full-time	92.1	60.6	94.3	77.7	95.7	88.7	90.6	71.7	89.8	55.3
Part-time	4.5	33.5	2.0	13.9	1.5	4.4	5.6	23.3	6.4	40.1
Involuntary part-time	3.5	5.9	3.7	8.3	2.7	6.9	3.8	5.0	3.9	4.7
**Irregular/unsocial hours** ^**1**^	3.8 (2.6)	2.6 (2.2)	3.7 (2.5)	2.7 (2.2)	4.1 (2.3)	2.8 (2.1)	3.5 (2.3)	2.6 (1.9)	4.0 (2.6)	2.5 (2.3)
**Lack of training**										
No	48.4	50.0	35.0	35.0	27.6	32.9	65.5	73.9	50.0	52.2
Yes	51.6	50.0	65.0	65.0	72.4	67.1	34.5	26.1	50.0	47.8
**Lack of representation**										
No	59.2	58.2	44.2	41.9	50.1	55.5	72.6	80.1	67.1	74.1
Yes	40.8	41.8	55.8	58.1	49.9	44.5	27.4	19.9	32.9	25.9
**Lack of participation** ^**1**^	6.0 (3.3)	6.2 (3.2)	5.7 (3.2)	5.9 (3.2)	7.4 (3.0)	7.4 (3.0)	4.8 (3.0)	4.8 (2.9)	5.5 (3.2)	5.8 (3.1)
**QUALITY OF INTRINSIC CHARACTERISTICS OF THE WORK TASK**										
**Low skill discretion** ^**1**^	3.8 (2.2)	4.3 (2.3)	4.9 (2.2)	5.5 (2.3)	4.6 (2.2)	4.8 (2.5)	3.6 (2.0)	3.7 (2.0)	3.9 (2.4)	3.8 (2.3)
**Low autonomy** ^**1**^	4.3 (2.7)	4.7 (2.6)	5.6 (2.5)	5.8 (2.4)	6.0 (2.9)	5.9 (2.8)	3.7 (2.3)	3.9 (2.1)	4.9 (2.7)	5.1 (2.3)
**High psy. demands** ^**1**^	5.4 (3.0)	5.5 (3.1)	5.3 (2.8)	5.3 (3.0)	4.1 (2.6)	4.2 (2.7)	5.3 (2.7)	5.7 (2.9)	5.5 (2.8)	5.9 (3.0)
**Lack of support**										
No	97.5	92.3	96.3	93.5	95.3	94.3	98.6	98.6	97.3	95.9
Yes	2.5	7.7	3.7	6.5	4.7	5.7	1.4	1.4	2.7	4.1
**Lack of career opportun.** ^**1**^	5.4 (3.0)	6.2 (3.1)	4.9 (2.6)	5.4 (2.6)	5.8 (2.6)	6.1 (2.6)	5.3 (2.6)	5.5 (2.7)	4.5 (2.7)	4.9 (2.8)

Employee population, 15–65 years old. ESS 2010.

^1^Mean with standard deviation in parentheses.

*Abbreviations:* *Contr*. contributory, *insuf. inc.* insufficient household income, *psy*. psychological, *opportun*. opportunities.

### Relation between work-related characteristics and poor mental well-being

The results of the regression analyses predicting poor mental well-being in employed men and women are presented in Table 4.

**Table 4:** **Estimates of the association between work-related health indicators and poor mental well-being in employed men and women (ESS 2010)** ^**a,b**^

	**Employed men**			**Employed women**			**Total**
	**Model 1**	**Model 2**	**Model 3**	**Model 1**	**Model 2**	**Model 3**	**Interaction Model 4**
	**Beta (S.E.)**	**Beta (S.E.)**	**Beta (S.E.)**	**Beta (S.E.)**	**Beta (S.E.)**	**Beta (S.E.)**	**Beta (S.E.)**
**Intercept**	2.057 (0.183)***	1.646 (0.184)***	1.358 (0.191)***	2.481 (0.197)***	2.036 (0.204)***	1.847 (0.210)***	1.458 (0.150)***
**Employment quality**							
Non-permanent contract	−0.110 (0.075)		−0.090 (0.074)	−0.069 (0.077)		−0.103 (0.076)	−0.130 (0.074)
Contributory earner with insufficient household income	0.696 (0.125)***		0.674 (0.123)***	0.907 (0.095)***		0.819 (0.094)***	0.651 (0.126)***
Main earner with insufficient household income	0.922 (0.080)***		0.801 (0.079)***	1.224 (0.103)***		1.117 (0.103)***	0.806 (0.080)***
Irregular and/or unsocial hours	0.053 (0.010)***		0.046 (0.010)***	0.072 (0.013)***		0.066 (0.013)***	0.055 (0.010)***
Part-time	0.109 (0.126)		0.134 (0.124)	0.228 (0.068)***		0.215 (0.067)***	0.138 (0.127)
Involuntary part-time	0.182 (0.133)		0.227 (0.131)	0.221 (0.111)*		0.159 (0.110)	0.202 (0.135)
Lack of training	0.033 (0.053)		−0.012 (0.052)	−0.130 (0.057)*		−0.186(0.057)***	−0.048 (0.053)
Lack of representation	0.297 (0.051)***		0.198 (0.051)***	0.169 (0.055)**		0.080 (0.056)	0.203 (0.052)***
Lack of participation	0.023 (0.008)**		0.010 (0.009)	0.029 (0.009)***		0.010 (0.010)	0.009 (0.009)
**Quality of intrinsic characteristics of the work task**							
Low skill discretion		0.033 (0.012)**	0.030 (0.012)*		0.049 (0.013)***	0.047 (0.013)***	0.039 (0.009)***
Low autonomy		0.007 (0.010)	−0.004 (0.011)		0.028 (0.011)**	0.012 (0.012)	0.004 (0.008)
High psychological demands		0.106 (0.009)***	0.096 (0.009)***		0.065 (0.009)***	0.053 (0.009)***	0.074 (0.006)***
Lack of co-workers support		0.407 (0.127)***	0.348 (0.126)**		0.298 (0.111)**	0.236 (0.110)*	0.294 (0.082)***
Lack of career opportunities		0.107 (0.009)***	0.095 (0.009)***		0.085 (0.010)***	0.074 (0.010)***	0.084 (0.007)***
**R** ^**2**^	0.062	0.074	0.099	0.061	0.045	0.080	0.090
**Interactions** ^**c**^							
Women* main earner with insufficient household income							0.270 (0.125)*

^a^*p ≤0.05; **p ≤0.01; ***p ≤0.001; ^b^Models are controlled for country dummies, age, education, children living at home and single parent; ^c^Only significant interactions are shown.

For men: Model 1 shows that having an insufficient household income while being a main (b=0.922; S.E.=0.080) or contributory earner (b=0.696; S.E.=0.125), having irregular and/or unsocial working hours (b=0.053; S.E.=0.010), a lack of representation (b=0.297; S.E.=0.051) and a lack of participation (b=0.023; S.E.=0.008) are positively associated with poor mental well-being. As expected, most indicators representing the intrinsic quality of the work task are associated with poor mental well-being (Model 2). When all indicators of employment quality are entered simultaneously with the indicators of the intrinsic quality of the work task (Model 3), most relations of employment quality with mental well-being remain significant. However, some estimates weaken a bit and the positive relation between lack of participation and poor mental well-being loses statistical significance.

For women: Having an insufficient household income while being a main (b=1.224; S.E.=0.103) or contributory earner (b=0.907; S.E.=0.095), having irregular and/or unsocial working hours (b=0.072; S.E.=0.013), part-time employment (b=0.228; S.E.=0.068), involuntary part-time employment (b=0.221; S.E.=0.111), a lack of participation (b=0.029; S.E.=0.009) and a lack of representation (b=0.169; S.E.=0.055) are positively associated with poor mental well-being. A significant negative association between a lack of training (b=−0.130; S.E.=0.057) and poor mental well-being is also found. The indicator set of the intrinsic quality of the work task shows a relationship with poor mental well-being. When all indicators of employment quality are entered simultaneously with the indicators of the quality of intrinsic characteristics of the work task in Model 3, the associations between involuntary part-time employment, lack of representation and lack of participation on the one hand and poor well-being on the other hand lose significance. Furthermore, the estimated effects of income, irregular and/or unsocial working hours and part-time employment weaken, while the effect of lack of training slightly increases.

Compared to the analyses for employed men, the model intercepts are much higher for employed women: female employees report a lower mental well-being, compared to their male counterparts (see Table 4). In both men and women, most indicators of less-favourable employment quality show a positive relation with poor mental well-being. However, for women a negative association between a lack of training and poor mental well-being is found. The explained variance of the final model for employed women (R^2^=0.085) is lower, compared to that of employed men (R^2^=0.101).

We look for additional support for gender differences in the vulnerability to low employment quality by probing into interaction effects (Model 4). Most interaction effects between the indicators of employment quality and gender were statistically insignificant, thus not supporting the differential vulnerability hypothesis. Only for being a main earner with insufficient household income we found support for a higher vulnerability to poor mental well-being in female compared to male wage-earners.

### Comparison between welfare regimes

Table 5 shows the regression analyses predicting poor mental well-being in employed men and women (Model 3) stratified by welfare regime.

**Table 5:** **Estimates of the association between work-related health indicators and poor mental well-being in employed men and women (ESS 2010)** ^**a, b**^

	**Traditional family**		**Southern European**		**Contradictory**		**Earner-carer**		**Market-oriented**	
	**Men**	**Women**	**Men**	**Women**	**Men**	**Women**	**Men**	**Women**	**Men**	**Women**
	**(n = 3,044)**	**(n = 2,785)**	**(n = 870)**	**(n = 811)**	**(n = 965)**	**(n = 938)**	**(n =340)**	**(n = 355)**	**(n = 852)**	**(n = 980)**
	**Beta (S.E.)**	**Beta (S.E.)**	**Beta (S.E.)**	**Beta (S.E.)**	**Beta (S.E.)**	**Beta (S.E.)**	**Beta (S.E.)**	**Beta (S.E.)**	**Beta (S.E.)**	**Beta (S.E.)**
**Intercept**	1.589 (0.238)***	1.593 (0.273)***	1.354 (0.321)***	2.569 (0.343)***	1.149 (0.455)*	1.351 (0.516)**	0.585 (0.561)	1.037 (0.590)	0.463 (0.468)	1.874(0.490)***
**Employment quality**										
Non-permanent contract	−0.405(0.115)***	−0.213 (0.122)	0.090 (0.159)	−0.092 (0.158)	0.198 (0.171)	0.027 (0.185)	0.266 (0.339)	−0.215 (0.294)	0.173 (0.242)	0.091 (0.208)
Contributory earner with insufficient household income	0.974 (0.203)***	1.069 (0.172)***	−0.397 (0.291)	0.704 (0.199)***	0.595 (0.226)**	0.670 (0.189)***	0.917 (0.844)	1.843 (0.665)**	1.027 (0.434)*	0.438 (0.226)
Main earner with insufficient household income	0.984 (0.119)***	1.386 (0.164)***	0.248 (0.161)	1.096 (0.250)***	0.598 (0.187)***	0.480 (0.221)*	1.657 (0.468)***	0.658 (0.519)	1.267 (0.261)***	1.227 (0.251)***
Irregular and/or unsocial hours	0.042 (0.015)**	0.068 (0.020)***	0.038 (0.024)	0.052 (0.030)	0.038 (0.029)	0.075 (0.035)*	0.009 (0.041)	0.036 (0.050)	0.074 (0.026)**	0.085 (0.032)**
Part-time	0.064 (0.167)	0.077 (0.094)	0.820 (0.409)*	0.813 (0.189)***	−0.044 (0.540)	0.239 (0.335)	−0.009 (0.385)	0.208 (0.222)	0.410 (0.273)	0.204 (0.152)
Involuntary part-time	0.337 (0.189)	0.023 (0.167)	0.272 (0.307)	0.383 (0.233)	0.607 (0.402)	0.372 (0.273)	−0.543 (0.480)	−0.324 (0.425)	−0.346 (0.344)	0.193 (0.326)
Lack of training	0.022 (0.073)	−0.275 (0.083)***	−0.059 (0.133)	−0.037 (0.140)	0.010 (0.157)	0.017 (0.165)	0.175 (0.192)	0.029 (0.214)	−0.198 (0.140)	−0.060 (0.144)
Lack of representation	0.137 (0.071)	0.090 (0.081)	0.250 (0.119)*	−0.078 (0.132)	0.264 (0.143)	−0.047 (0.143)	0.135 (0.200)	−0.188 (0.227)	0.180 (0.144)	0.430 (0.154)**
Lack of participation	0.006 (0.012)	0.012 (0.014)	−0.043 (0.023)	−0.013 (0.024)	0.042 (0.028)	0.029 (0.028)	0.067 (0.033)*	0.052 (0.034)	0.042 (0.026)	0.017 (0.025)
**Quality of intrinsic characteristics of the work task**										
Low skill discretion	0.036 (0.018)*	0.058 (0.020)**	0.000 (0.029)	−0.022 (0.032)*	0.025 (0.034)	0.042 (0.034)	0.071 (0.049)	0.070 (0.052)	0.026 (0.032)	0.068 (0.034)*
Low autonomy	−0.009 (0.016)	0.038 (0.017)*	0.072 (0.031)*	0.007 (0.034)	−0.032 (0.030)	−0.018 (0.029)	0.039 (0.045)	0.012 (0.046)	−0.061 (0.032)	−0.039 (0.033)
High psychological demands	0.076 (0.012)***	0.045 (0.013)***	0.048 (0.023)*	0.060 (0.022)**	0.118 (0.027)***	0.082 (0.026)**	0.134 (0.034)***	0.084 (0.033)*	0.198 (0.023)***	0.046 (0.024)
Lack of co-workers support	0.521 (0.175)**	0.319 (0.151)*	−0.699 (0.308)*	−0.513 (0.261)*	0.492 (0. 309)*	0.688 (0.292)*	0.600 (0.721)	0.506 (0.764)	0.475 (0.398)	0.204 (0.335)
Lack of career opportunities	0.094 (0.013)***	0.076 (0.014)***	0.099 (0.023)***	0.083 (0.026)**	0.109 (0.029)***	0.124 (0.029)***	0.056 (0.037)	0.085 (0.036)*	0.087 (0.026)***	0.015 (0.026)
**R** ^**2**^	0.096	0.094	0.109	0.116	0.114	0.101	0.197	0.143	0.175	0.086
**Interaction model 4** ^**c**^	**Traditional family**		**Southern European**		**Contradictory**		**Earner-carer**		**Market-oriented**	
Women* contributory earner with insufficient household income				0.956 (0.344)**						
Women* main earner with insufficient household income				0.773 (0.283)**						
Women* lack of training		−0.224 (0.106)*								
Women* lack of representation				−0.360 (0.173)*						

^a^*p ≤0.05; **p ≤0.01; ***p ≤0.001; ^b^Models controlled for country dummies, age, education, children living at home and single parent; ^c^Only significant interactions shown.

For men: In all but the Southern European welfare regime, employed men show a significant relation between poor mental well-being and indicators of (insufficient) income. In Southern European countries, employed men report an association of lack of representation and part-time employment with mental well-being. In Traditional family and Market-oriented welfare regimes, irregular and/or unsocial working hours are positively associated with poor mental well-being. Further, a positive association between lack of participation and poor mental well-being is found in the Earner-carer welfare regime. In the Traditional family welfare regime, poor mental well-being is negatively associated with holding a non-permanent contract.

For women: In all welfare regimes, employed women’s mental well-being shows a relation with the indicators of income. In the Traditional family, Contradictory and Market-oriented welfare regimes, irregular and/or unsocial working hours are positively associated with poor mental well-being. In the Southern European welfare regime, part-time employment is positively associated with poor mental well-being. In the Market-oriented welfare regime, a lack of representation is positively associated with poor mental well-being.

For both genders in all welfare regimes, at least one sub-dimension of employment quality is found to be associated with poor mental well-being, after statistically controlling for the quality of intrinsic characteristics of the work task. We look for additional support for gender differences in the vulnerability to low employment quality across welfare regimes by probing into interaction effects (Table 5, Model 4). Across all welfare regimes most interaction effects between the indicators of employment quality and gender were statistically insignificant. However, men’s well-being is more vulnerable to a lack of representation compared to women’s well-being in the Southern European welfare regime. Furthermore, in the Southern European welfare regime, women’s well-being is more vulnerable to having an insufficient household income while being a main or contributory earner than men’s well-being. In the Traditional family welfare regime women’s well-being is less vulnerable to a lack of training, compared to men’s well-being.

## Discussion

The aim of this study was threefold. First, we aimed to investigate whether a set of indicators referring to employment quality is associated with the mental well-being of employees, even when controlling for the intrinsic quality of the work task. We represented employment quality using a multidimensional approach. The second aim was to investigate whether the relation between employment quality and mental well-being, controlled for the quality of intrinsic characteristics of the work task, differs for men and women. Thirdly, we expected that welfare regimes are (partially) mediating this differential vulnerability, e.g. through policies facilitating work-family reconciliation.

### Relation between work-related characteristics and poor mental well-being

In general, the results of this study are demonstrating the added value of employment quality in understanding employee mental well-being across Europe. We found that most indicators of low employment quality and poor mental well-being are positively related to each other, even when controlling for the intrinsic quality of the work task.

### Gender differences

For both men and women, at least one sub-dimension of low employment quality is significantly related with poor mental well-being. This shows that both men and women’s mental well-being suffers from low employment quality. Nevertheless, differences between gender groups are also found. The gender-stratified models point in the direction of a higher vulnerability of women to low employment quality. However, these findings were only partially supported by the interaction models. The interaction models (especially those across welfare regimes) probably do not have sufficient power to detect these effects.

### Comparison between welfare regimes

This study demonstrated that the differential vulnerability of men and women to bad quality employment is partly explained by welfare regimes. We found that women’s well-being is more vulnerable to an insufficient household income when being the main earner in the household especially in Traditional family and Southern European countries. One possible explanation for these observations is that women are typically found to be the main responsible for homemaking, while receiving less support for combining the employee and homemaker role in these countries [27]. Less generous family benefits and services could explain why women are more vulnerable to an insufficient household income while being the main earner.

Furthermore, employed women from Traditional family, Contradictory and Market-oriented countries report a stronger positive relation between irregular and/or unsocial working hours and poor mental well-being, compared to their male counterparts. This could be due to lower (institutional) support for balancing work and family demands in the above-described countries since intense and unsocial hours have a negative impact on balancing work and family demands [39].

Remarkably, a lack of training is negatively associated with poor mental well-being in women in the Traditional family countries. The absence of training opportunities may be perceived as avoiding an increasing work-life conflict in the Traditional family welfare regime.

Part-time employment is positively associated with poor female mental well-being in Southern European countries. This finding could be explained in the context of the financial crisis. In the Southern European countries, family economic needs may have pushed women, who were initially full-time caregivers, into the labour market in order to guarantee a second income. In such a scenario, their employment adds to their greater domestic workload in the context of both minimal public childcare support and men’s limited contribution to housework [40].

In Market-oriented countries, employed women report a positive relation between poor mental well-being and lack of representation. We found that in Market-oriented countries, employed women are more likely than employed men to be trade union members. Furthermore, in Market-oriented countries – even more than in other types of countries – trade union members are a specific group of people holding high-quality jobs and/or jobs in the public sector [41]. The highest level of employer-supported childcare is seen in the public sector [41]. This could explain why women’s mental well-being in Market-oriented countries is more vulnerable to a lack of representation. The availability of employer-supported childcare at work-sites may be stimulated by the presence of trade union representation. In the context of very low government support for work-family reconciliation, employer-supported childcare is a very valuable asset.

Unlike employed women, employed men experience a positive association between a lack of representation and poor mental well-being in Southern European countries. This finding might reflect the different types of jobs or sectors that men and women mostly work in. However, this might also reflect the meaning men in Southern European welfare regimes attribute to employee representation. Individuals attribute meaning to objects and events, and they do so within their historical and cultural context [23]. Historically and culturally throughout the ‘traditional family’ model in Southern European welfare regime, the labour role of men is key to their identity, while the labour role of women is often secondary to their caring/parenting role. As men see themselves more unified with their job, bargaining power at the work place is more meaningful to them. The lack of bargaining power can convey as a symbol of low status and worth, which can adversely affect a person’s self-esteem and dignity. These internalized feelings may lead to poorer mental well-being [23].

In the Traditional family welfare regime there is a negative association between holding a non-permanent contract and poor mental well-being in employed men. The ‘traditional family’ model may also account for part of the explanation. The absence of a permanent contract may be perceived as a factor endangering the traditional male breadwinner role.

### Strengths and limitations

Our study has some limitations because of the use of secondary data. Firstly, the data is derived from a cross-sectional sample so we cannot formally establish the causal direction of the relationships under study. Furthermore, we cannot exclude reverse causation: that is people with poorer mental health could be more likely to find employment in lower quality work environments [42]. Secondly, the indicators of employment quality are only proxies for the underlying theoretical concepts. For instance, one of the sub-dimensions of the employment conditions is income and rights. ESS data lack measures of social rights and additional benefits (e.g. paid overtime, additional sickness insurance, etc.) and hence, only income is included from this dimension. Thirdly, the dataset used for this study in terms of mental well-being only contained three of the original five items of the WHO-5 Well-being Index [35]. However, since the internal consistency of the Well-being index has proven to be excellent, we are confident that the use of a three-item scale does not distort our results [43]. Moreover, the ESS is a large source of reliable cross-national European data, which was supplemented in 2010 with a module on work, family and well-being, making it a database that is particularly apt for investigating our research questions.

The most important strength of this study is that it goes beyond standard measures of the psychosocial work environment and complements these with multiple indicators of employment quality. Our results suggest that future research should include all aspects of the ‘changed world of work’, when examining its effect on employee mental well-being. Moreover, our analyses point to the added value of gender-stratified analyses and estimating interactions between gender and employment quality when investigating the effect of employment quality on mental well-being. Gender-stratified analyses have already proven to be useful in mental health-related unemployment research [44].

## Conclusions

From a policy perspective, the results suggest that governments should safeguard high-quality employment arrangements for all employees regardless of gender. Furthermore, policy models balancing family life and work (cf. Earner-carer countries) were the most worker-friendly models. In welfare regimes with family-life-and-work-balancing policies, employee mental well-being is less susceptible to low employment quality in both men and women, pointing at the importance of such policies for gender equality in mental well-being and employee mental well-being in general.
